# Expression profiling on subclasses of primary parotid gland carcinomas

**DOI:** 10.18632/oncotarget.27797

**Published:** 2020-11-10

**Authors:** Jeannine Meinrath, Anja Haak, Nesrin Igci, Priya Dalvi, Christoph Arolt, Sonja Meemboor, Udo Siebolts, Hannah Eischeidt-Scholz, Claudia Wickenhauser, Inga Grünewald, Uta Drebber, Reinhard Büttner, Alexander Quaas, Jens-Peter Klußmann, Margarete Odenthal, Dirk Beutner, Moritz Meyer

**Affiliations:** ^1^Institute of Pathology, University Hospital of Cologne, Cologne, Germany; ^2^Department of Pathology, University of Halle, Halle, Germany; ^3^Center for Molecular Medicine, University of Cologne, Cologne, Germany; ^4^Center of Integrative Oncology, University Hospital of Cologne, Cologne, Germany; ^5^Department of Pathology, University Hospital of Münster, Münster, Germany; ^6^Department of Otorhinolaryngology, Head and Neck Surgery, University of Cologne, Cologne, Germany; ^7^Department of Otorhinolaryngology, Head and Neck Surgery, University Medical Center Göttingen, Göttingen, Germany; ^8^Department of Otorhinolaryngology, Head and Neck Surgery, University Hospital Essen, University Duisburg-Essen, Duisburg, Germany

**Keywords:** cancer, salivary gland, nanostring, pancancer pathway, gene expression

## Abstract

Introduction: The underlying molecular mechanisms of parotid gland carcinomas (PGC) are still unknown. Knowledge about the tumor-driving signaling pathways is necessary either for diagnostics or developing new therapeutic options in this heterogeneous and rare entity.

Material and Methods: 94 matching RNA formalin-fixed and paraffin-embedded tissue samples from PGC and the corresponding non-tumor area, RNA quality and quantity were sufficient for gene expression profiling of 770 genes using the NanoString's nCounter technology. Oncogenic and tumor suppressor genes were examined in the three common PGC tumor entities: adenoid cystic carcinoma (ACC), adenocarcinoma NOS (AC-NOS), and mucoepidermoid carcinoma (MEC).

Results: Expression profiling and subsequent hierarchical cluster analysis clearly differentiated between non-tumor gland tissue samples and PGC. In addition expression pattern of all three entities differed. The extensive pathway analysis proved a prominent dysregulation of the Wnt signaling pathway in the three PGC entities. Moreover, transcript upstream analysis demonstrated a pronounced activation of the PI3K pathway in ACC and MEC.

Discussion: Our findings revealed divergent molecular expression profiles in MEC, ACC and AC-NOS that are presently studied for their potential application in PGC diagnostics. Importantly, identification of Wnt and PI3K signaling in PGC revealed novel options of PGC therapy.

## INTRODUCTION

Salivary gland carcinomas account for 1–5% of all head and neck tumors worldwide and, according to the World Health Organization (WHO), can be divided into a total of 22 subgroups [1–3]. Despite their lower incidence compared to other carcinomas, they represent a scientifically interesting and little-researched tumor group to date due to their diversity of entities. The most frequent histological types are adenocarcinomas, adenoid cystic carcinomas and mucoepidermoid carcinomas [4]. The anatomical location and the potential local spreading pattern of salivary gland cancer (SGC)—especially the parotid gland (PGC)—make these tumors, on the one hand, surgically demanding, and on the other the psychosocial role for the patient quite vital, given that the surgery may involve extensive cosmetic changes to the patient’s face. The development of salivary gland tumors is still largely unexplained.

The treatment of first choice for salivary gland malignancies is surgical tumor excision. Depending on the degree of differentiation, primary tumor extent (T classification) and lymph node status (N classification), surgical therapy should include a more-or-less extensive neck dissection [5]. Adjuvant radio (chemo) therapy is additionally recommended in patients with high T classification, positive resection margins, pN+, perineural invasion or a high-grade subtype, especially in MEC. The benefit of additional chemotherapy has not been conclusively clarified, the main reason being, in particular, the lack of data on effectiveness. There are only limited studies on systemic therapy concepts that could be considered in palliative situations [6–8].

Recently, the developments in molecular pathology diagnostics open up manifold possibilities, which provide new diagnostic, prognostic and especially therapeutic approaches as well as points of targeted therapy. Due to the overall lower incidence compared to other tumors, the more precise molecular examination of tumor DNA—and especially the tumor RNA—is a hurdle, as an eventual therapeutic relevance is not determinable due to the often- missing large cohorts of SGC [9, 10]. Over the past decade, molecularly targeted therapies that block important oncogenic pathways have made remarkable progress [11]. A personalized tumor therapy concept—as applied in the case of colon or lung carcinomas—is not yet established, due to limited data of tumor driving pathways, which could then be targeted by future individualized therapy approaches [12–14]. Recent study demonstrated high frequencies of activating *PIK3CA* and *HRAS* mutations as well as deactivating *TP53* mutations in epithelial myoepithelial, salivary duct, squamous cell, oncocytic and in large cell undifferentiated carcinoma as well as in basal cell adenocarcinoma [15]. However, these tumor driving genetic alterations were not found in mucoepidermoid (MEC) and adenoid cystic (ACC) parotid gland carcinoma or on adenocarcinoma not otherwise specified (AC-NOS). On the one hand, these entities should be investigated due to their frequency and the hitherto poorly understood tumor genesis. Novel diagnostic marker genes and therapeutic targets for these entities are urgently needed.

Therefore, we focused on these entities and compiled a sample collective from a total of 61 PGC patients. Importantly, comprehensive expression profiling on histologically well-classified MEC, ACC, and AC-NOS carcinoma defined divergent expression patterns between the entities. Moreover, we studied tumor driving pathways and demonstrated a common activation of the PI3K/AKT pathway. Most notably, prominent stimulation of Wnt signaling was observed in most MEC, ACC, and AC-NOS.

## RESULTS

### Clinical characteristics

To access the deregulation of gene expression in the most frequent parotid gland carcinoma entities, MEC, ACC, and AC-NOS, we collected 61 PGC samples and the corresponding non-tumor tissues. Macrodissection and RNA extraction were performed from all matching FFPE samples but there was only sufficient RNA available from 47 matching samples for expression profiling from both, tumor tissue and normal tissue. Clinicopathological characteristics of the 47 patients, considered in our study, are summarized in Table 1.

**Table 1:** Overview of the clinical characteristics of the included patients

Tumor entity	Number	Gender		T-classification			N-classification		
	(*n*)	Male	Female	pT1/ pT2	pT3/ pT4	pT n. a.	pN0	pN1/ pN2	pN n. a.
**Mucoepidermoid carcinoma**	13	2	11	9	3	1	10	2	1
**Adenoid cystic carcinoma**	14	5	9	5	8	1	10	3	1
**Adenocarcinoma NOS**	20	12	8	4	16	0	7	13	0

### Genetic differences within tumor entities in regard to gene expression

**Figure 1:**
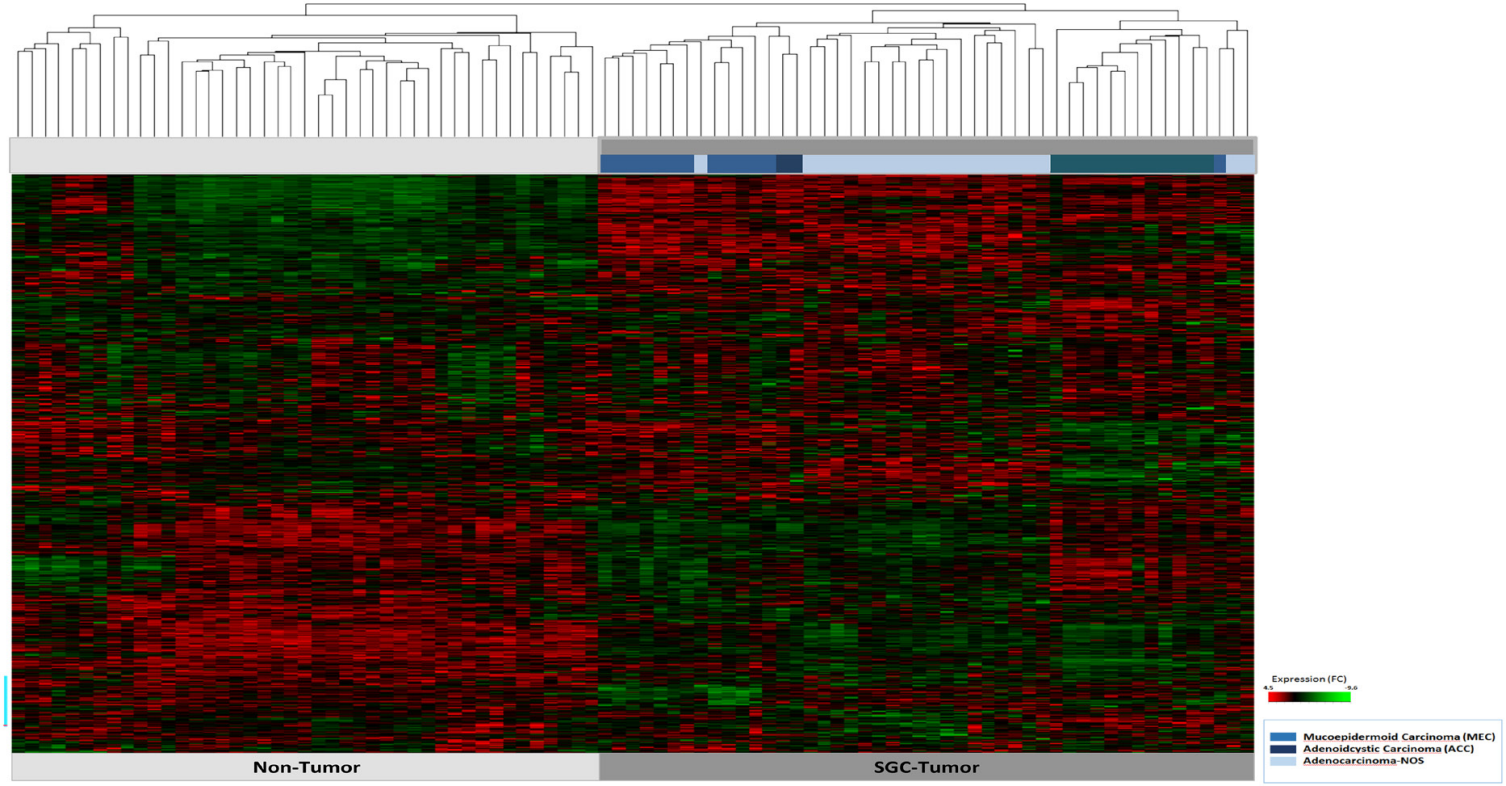
Hierarchical cluster analysis of all 47 patient samples of tumor tissue (*n* = 47) and corresponding non-tumor tissue (*n* = 47). The tumor and non-tumor samples (*n* = 94) are arranged on the x-axis, the transcript assignment on the y-axis. The entities are differentiated by the bars in blue tones (see legend). A high gene expression is shown in the heatmap red, a downregulated expression in green.

**Figure 2:**
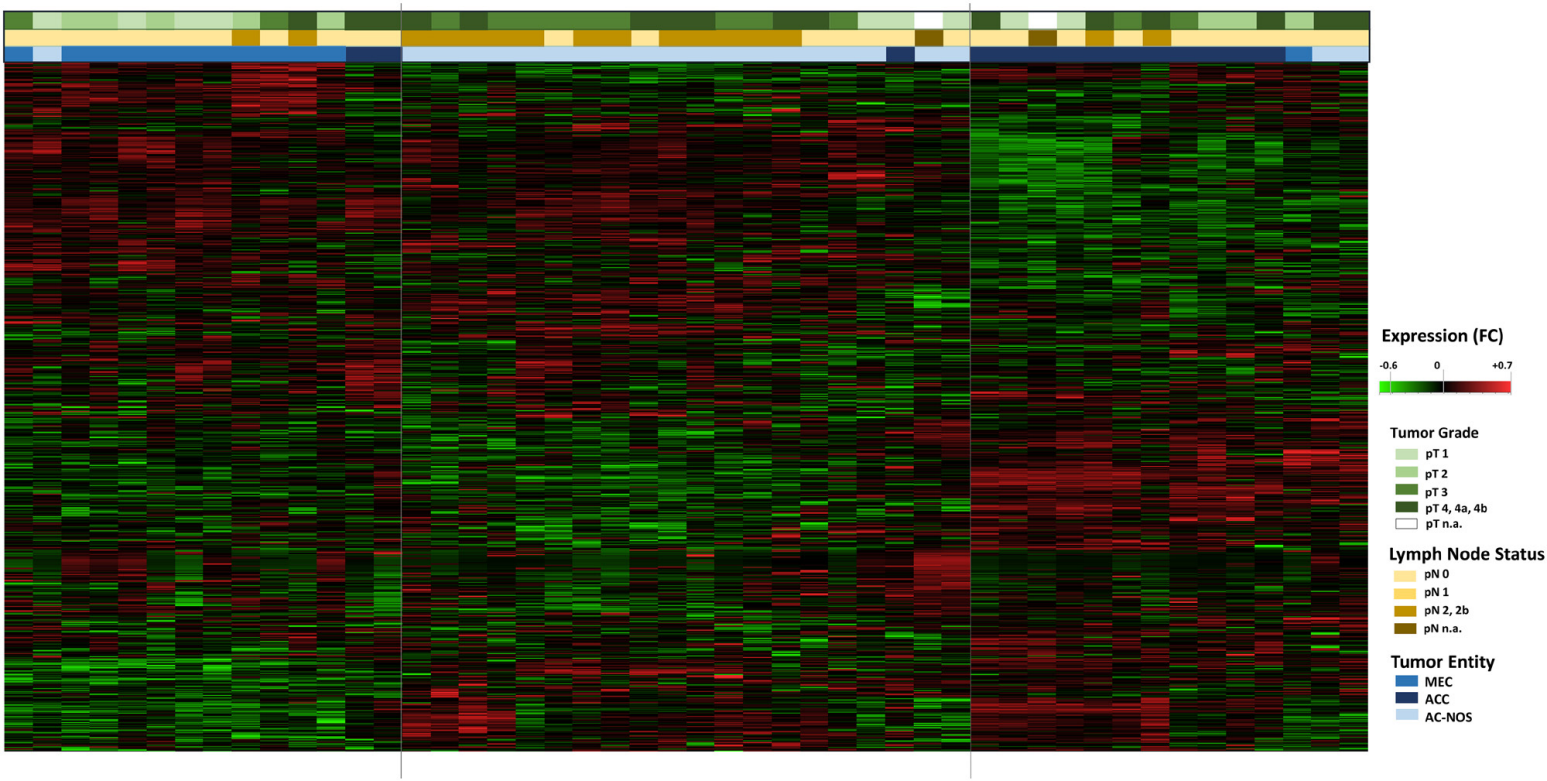
Hierarchical cluster Heatmap Analysis of tumor entities with tumor growth (pT) and lymphogenic metastasis (pN): All tumor samples of the 47 patients are shown (*n* = 47, x-axis). The transcript assignment is on the y-axis. The upper color bars represent the tumor size (pT, in green tones), the pathological lymph node status (pN, in brown tones) and the tumor entities (in blue tones). An increased gene expression is shown in the heatmap red, a decreased gene expression in green.

A total of 730 highly cancer relevant genes involved in 13 tumor driving signaling pathways were studied (Supplementary Table 2). Hierarchical cluster analysis showed a clear difference in the expression pattern of the cancer associated, in comparison to the non-tumor associated, gene sets (Figure 1). Moreover, we observed entity specific gene clusters. In particular, in ACC tumors distinct gene expressions were shown (Figure 1). Detailed comparison of the expression profiles of MEC, ACC, and AC-NOS confirmed that the differences in expression clusters of ACC in comparison to MEC and AC-NOS (Supplementary Figure 1A–1C), whereas MEC and AC-NOS differ only slightly from each other (Figure 2, Supplementary Figure 1D–1E). Importantly in ACC, genes of inflammatory pathways were upregulated such as *TNF1* as well as *interleukin 1* and *6* and mediators (e.g., *IRAK 2*, *3*, *IL1B*, *JAK3*, *STAT1*, *STAT4*) (Supplementary Figure 1C). In MEC and AC-NOS, for example, the subunits of *phospholipase C*, *D*, *FGF12* and the chemokine *CXXC4* were highly expressed (Supplementary Figure 1E).

In a further hierarchical cluster analysis, the tumor entities were examined in detail, including primary tumor progression (pT) and locoregional lymph node metastasis (pN). Figure 2 shows the differences in gene expression patterns between the entities MEC, ACC and AC-NOS. With the exception of a few individual samples, the samples were grouped according to their entities and gene expression profiles. A grouping due to tumor progression or lymphogenic metastasis across the entities was not apparent. The grouping was based on similarities in gene expression was therefore based more on membership of an entity than on the degree of progression or metastasis.

It should also be noted that these tumors generally have a higher T and N classification.

### Tumor-relevant gene expression signature in mucoepidermoid carcinoma

**Figure 3:**
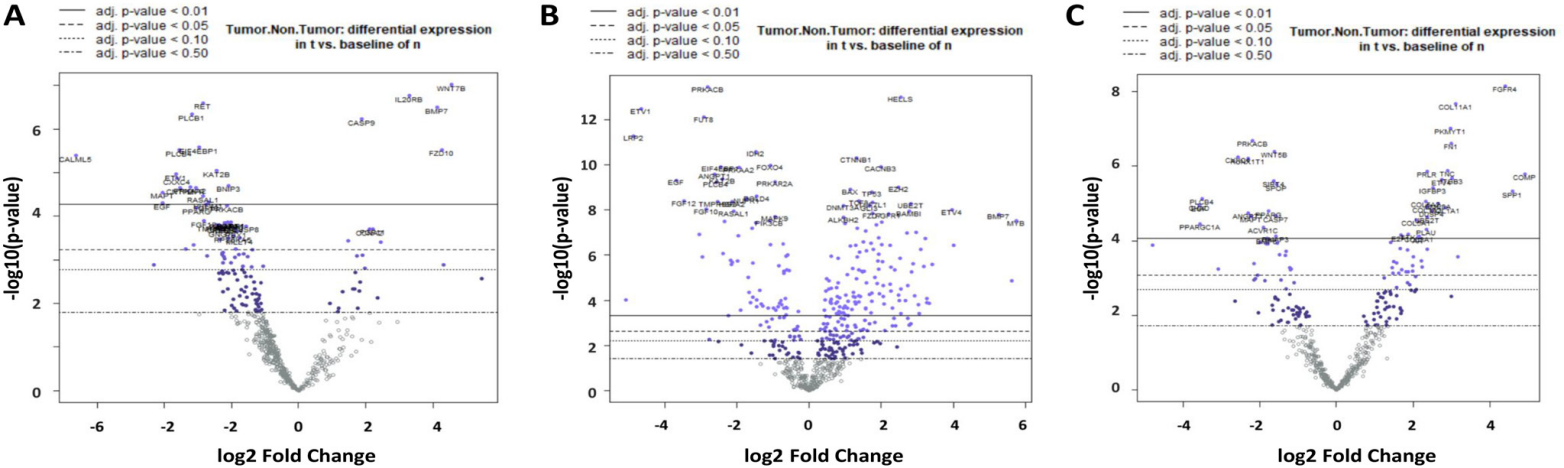
Volcano plot of differential gene expression in MEC (**A**), ACC (**B**) and AC-NOS (**C**). On the X-axis there is log2 fc (fold change), on the Y-axis the -log10 *p*-value. The divergent expression changes tumor vs. non-tumor with the highest significance after the Benjamin Hochberg correction (adjusted *p*-value < 0.01) are located above the continuous line.

The divergent gene expression pattern between tumor and non-tumor area of the mucoepidermoid carcinomas shows, as demonstrated in Figure 3A and Table 2A, a clear upregulation of *COMP*. Similarly, some Wnt pathway genes such as *WNT7B*, *FZD10* and *SFRP4* are significantly overexpressed, whereas *WIF1*, *CXXC4*, *PLCB1 and 4* are downregulated. Upregulation is also found in matrix proteins such as *COL1A1* and *COL11A1*.

**Table 2:** Vulcano-plot analysis

A) MEC			
Gene	log2 fc	*p*-value	signiling pathway
COMP	5,44	0,00272	PI3K
WNT7B	4,54	9,62E-08	Hedgehog, Wnt
COL1A1	4,31	0,00129	PI3K
FZD10	4,27	0,00000298	Wnt
BMP7	4,13	0,000000317	TGF-beta
IL20RB	3,29	1,73E-07	JAK-STAT
MMP9	2,95	0,0269	Transcriptional Misregulation
FGFR3	2,44	0,0004	Driver gene, MAPK, PI3K, Ras
SPP1	2,4	0,0278	PI3K
COL11A1	2,35	0,00753	PI3K
FGF11	2,22	0,00019	MAPK, PI3K, Ras
SFRP4	2,17	0,077	Wnt
CCR7	2,12	0,0169	Transcriptional Misregulation
CCNA2	2,12	0,0002	Cell Cycle - Apoptosis
SFN	1,98	0,00158	Cell Cycle - Apoptosis
EIF4EBP1	−2,97	2,65E-06	PI3K
PPARG	−3,04	6,38E-05	Transcriptional Misregulation
LIFR	−3,06	2,25E-05	JAK-STAT
ITGA7	−3,13	0,00045	PI3K
PLCB1	−3,19	4,53E-07	Wnt
PRKAA2	−3,23	2,15E-05	PI3K
GADD45G	−3,36	0,00056	Cell Cycle - Apoptosis
CNTFR	−3,53	2,25E-05	JAK-STAT
PLCB4	−3,55	3,02E-06	Wnt
CXXC4	−3,63	1,33E-05	Wnt
ETV1	−3,65	1,09E-05	Transcriptional Misregulation
MAPT	−4,05	2,82E-05	MAPK
EGF	−4,06	4,97E-05	MAPK, PI3K, Ras
WIF1	−4,31	0,00131	Wnt
CALML5	−6,64	4,00E-06	Ras

**B) ACC**			
**Gene**	**log2 fc**	***p*-value **	**signiling pathway**
COMP	4,53	0,00072	PI3K
BMP7	4,1	5,41E-07	TGF-beta
MYB	4,01	0,00034	PI3K
ETV4	3,54	1,45E-06	Transcriptional Misregulation
AMH	3	6,72E-05	TGF-beta
CACNA2D2	2,3	4,73E-03	MAPK
FANCA	2,23	4,34E-05	DNA Damage - Repair
COL5A1	2,12	3,67E-03	PI3K
PKMYT1	2,12	1,20E-04	Cell Cycle - Apoptosis
BMP8A	1,99	3,80E-03	TGF-beta
COL2A1	1,98	1,34E-02	PI3K
ITGB6	1,72	1,97E-02	PI3K
CLCF1	1,71	7,21E-02	JAK-STAT
COL5A2	1,62	3,56E-02	PI3K
LAMC2	1,59	1,23E-02	PI3K
MAPT	−3,55	3,20E-09	MAPK
PLCB4	−3,61	1,19E-08	Wnt
FGF10	−3,62	4,33E-08	MAPK, PI3K, Ras
KAT2B	−3,64	2,40E-09	Notch
ANGPT1	−3,75	6,45E-09	PI3K, Ras
FGF22	−3,84	4,39E-09	MAPK, PI3K, Ras
TMPRSS2	−3,86	5,28E-11	Transcriptional Misregulation
PRKACB	−3,98	3,50E-12	Cell Cycle - Apoptosis, Hedgehog, MAPK, Ras, Wnt
FUT8	−3,99	3,88E-10	Transcriptional Misregulation
WNT5A	−4,34	3,58E-09	Hedgehog, Wnt
EGF	−4,51	9,47E-10	MAPK, PI3K, Ras
EGF12	−4,78	1,57E-11	MAPK, PI3K, Ras
ETV1	−5,88	9,31E-13	Transcriptional Misregulation
LRP2	−5,97	1,67E-13	Hedgehog
WIF1	−6,21	2,00E-06	Wnt

**C) AC-NOS**			
**Gene**	**log2 fc**	***p*-value **	**signiling pathway**
COMP	4,91	1,61E-06	PI3K
SPP1	4,6	4,76E-06	PI3K
FGFR4	4,41	7,09E-09	MAPK, PI3K, Ras
ITGB6	3,16	2,70E-04	PI3K
COL11A1	3,11	2,06E-08	PI3K
ITGB3	3,02	2,12E-06	PI3K
MMP9	3	3,09E-03	Transcriptional Misregulation
FN1	3	2,39E-07	PI3K
PKMYT1	2,97	9,42E-08	Cell Cycle – Apoptosis
TNC	2,9	1,33E-06	PI3K
COL1A1	2,84	1,28E-05	PI3K
ETV4	2,74	2,29E-06	Transcriptional Misregulation
BMP8A	2,65	9,81E-06	TGF-beta
IGFBP3	2,52	3,37E-06	Transcriptional Misregulation
DUSP4	2,49	1,56E-05	MAPK
ETV1	-2,12	1,05E-03	Transcriptional Misregulation
CACNB2	-2,15	4,10E-04	MAPK
RASAL1	-2,16	1,13E-03	Ras
PRKACB	-2,19	2,04E-07	Cell Cycle - Apoptosis, Hedgehog, MAPK, Ras, Wnt
MAPT	-2,24	2,21E-05	MAPK
ANGPT1	-2,31	1,79E-05	PI3K, Ras
RUNXIT1	-2,31	6,20E-07	Transcriptional Misregulation
CXXC4	-2,58	5,63E-07	Wnt
SFRP1	-2,66	4,18E-03	Wnt
LRP2	-3,09	5,70E-04	Hedgehog
PLCB4	-3,51	7,49E-06	Wnt
PPARGC1A	-3,57	3,57E-05	Chromatin Modification
CHAD	-3,58	1,10E-05	PI3K
EGF	-3,59	1,14E-05	MAPK, PI3K, Ras
WIF1	-4,81	1,30E-04	Wnt

**Figure 4:**
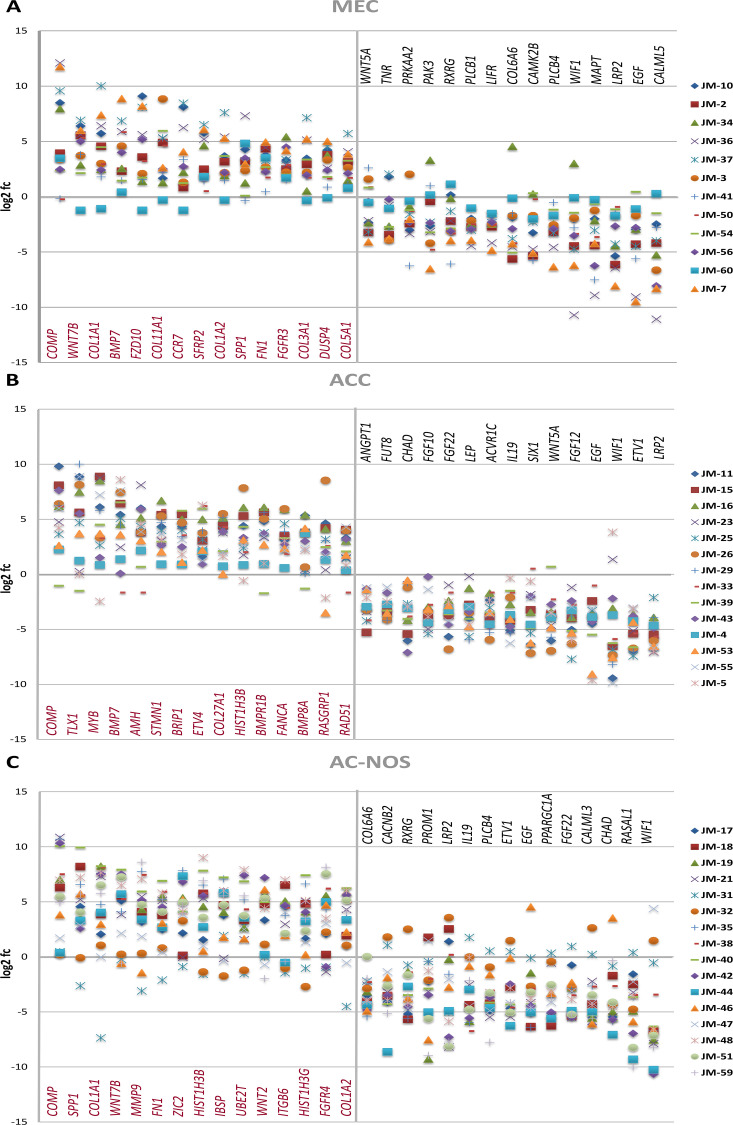
Gene expression of MEC (**A**), ACC (**B**) and AC-NOS (**C**) of individual tumor patient samples compared to their corresponding non-tumor sample expressed as log2 fc (fold change Tumor vs Non-Tumor). Patient samples (JM No.) are represented by colored markings (see legend).

As shown in Figure 4A, the analysis of the corresponding tumor and non-tumor samples also revealed upregulation of *COMP*, matrix proteins such as *COL5A1*, *COL11A1*, and *FN1*, and genes of the Wnt pathway (*WNT7B*, *FZD10*, *SFRP2*), while *PLCB1*, *CAMK2B*, *PLCB4* and *WIF1* are downregulated (Table 3A).

**Table 3:** Pair-to-pair analysis

A) MEC			
Gene	log2 fc	*p*-value	signiling pathway
COMP	22,82	0,00032522	PI3K
WNT7B	4,42	0,00000038	Hedgehog, Wnt
COL1A1	30,76	0,00004253	PI3K
BMP7	6,91	0,00000031	TGF-beta
FZD10	6,9	0,0000024	Wnt
COL11A1	16,69	0,00000789	PI3K
CCR7	7,28	0,00044422	Transcriptional Misregulation
SFRP2	11,79	0,00042547	Wnt
COL1A2	12,99	0,00003073	PI3K
SPP1	7,34	0,00082186	PI3K
FN1	9,31	0,00015914	PI3K
FGFR3	9,66	0,00000063	Driver Gene, MAPK, PI3K, Ras
COL3A1	11,29	0,00015946	PI3K
DUSP4	9,57	0,00000154	MAPK
COL5A1	8,48	0,00006621	PI3K
WNT5A	-2,24	0,12500988	Wnt
TNR	-1,02	0,3388007	PI3K
PRKAA2	-3,79	0,00071002	PI3K
PAK3	-1,75	0,01448371	Ras
RXRG	-1,32	0,1345256	Transcriptional Misregulation
PLCB1	-5,35	0,00000284	Wnt
LIFR	-6,4	0,00000095	JAK-STAT
COL6A6	-1,07	0,74636859	PI3K
CAMK2B	-1,89	0,01919538	Wnt
PLCB4	-7,97	0,00000867	Wnt
WIF1	-7,23	0,00811211	Wnt
MAPT	-5,03	0,00023095	MAPK
LRP2	-3	0,00303356	Hedgehog
EGF	-6,71	0,00051303	MAPK, PI3K, Ras
CALML5	-18,29	0,0000097	Ras

**B) ACC**			
**Gene**	**log2 fc**	***p*-value **	**signiling pathway**
COMP	11,85	0,00005072	PI3K
TLX1	9,68	0,00027461	Transcriptional Misregulation
MYB	14,63	0,00005751	PI3K
BMP7	12,37	0,00001101	TGF-beta
AMH	4,73	0,0000381	TGF-beta
STMN1	13,26	0,00000004	MAPK
BRIP1	7,13	0,00000001	DNA Damage – Repair
ETV4	6,45	0,00000302	Transcriptional Misregulation
COL27A1	9,98	0,00000016	PI3K
HIST1H3B	8,99	0,00000451	Driver Gene, Transcriptional Misregulation
BMPR1B	8,81	0,00015217	TGF-beta
FANCA	5,1	0,00000001	DNA Damage - Repair
BMP8A	2,64	0,00296188	TGF-beta
RASGRP1	4,31	0,00101834	MAPK, Ras
RAD51	1,39	0,00652304	DNA Damage - Repair
ANGPT1	−6,72	0,00000003	PI3K, Ras
FUT8	−8,03	0,00000001	Transcriptional Misregulation
CHAD	−6,3	0,00006851	PI3K
FGF10	−5,6	0,00000005	MAPK, PI3K, Ras
FGF22	−2,82	0,00007386	MAPK, PI3K, Ras
LEP	−2,08	0,00080883	JAK-STAT
ACVR1C	−1,78	0,00235731	TGF-beta
IL19	−1,71	0,00470287	JAK-STAT
SIX1	−8,42	0,00000773	Transcriptional Misregulation
WNT5A	−13,69	0,00000014	Hedgehog, Wnt
FGF12	−9,87	0,00000001	MAPK, PI3K, Ras
EGF	−12,82	0,00000001	MAPK, PI3K, Ras
WIF1	−17,79	0,00053199	Wnt
ETV1	−31,74	0,00000001	Transcriptional Misregulation
LRP2	−9,36	0,00000001	Hedgehog

**C) AC-NOS**			
**Gene**	**log2 fc**	***p*-value **	**signiling pathway**
COMP	16,8	0,00000001	PI3K
SPP1	10,39	0,00000001	PI3K
COL1A1	7,02	0,00000001	PI3K
WNT7B	3,26	0,00000001	Hedgehog, Wnt
MMP9	4,48	0,00007157	Transcriptional Misregulation
FN1	7,85	0,00000001	PI3K
ZIC2	1,66	0,00045604	Hedgehog
HIST1H3B	6,5	0,00000001	Driver Gene, Transcriptional Misregulation
IBSP	1,61	0,00140148	PI3K
UBE2T	4,63	0,00000001	DNA Damage - Repair
WNT2	1,7	0,00000115	Wnt
ITGB6	6,13	0,00000001	PI3K
HIST1H3G	4,92	0,00000001	Driver Gene, Transcriptional Misregulation
FGFR4	2,02	0,0000605	MAPK, PI3K, Ras
COL1A2	6,07	0,00000001	PI3K
COL6A6	−1,25	0,04340979	PI3K
CACNB2	−4,42	0,00000001	MAPK
RXRG	−1,37	0,00009869	Transcriptional Misregulation
PROM1	−3,11	0,000004	Transcriptional Misregulation
LRP2	−4	0,00000001	Hedgehog
IL19	−1,41	0,00010192	JAK-STAT
PLCB4	−7,6	0,00000001	Wnt
ETV1	−11,53	0,00000001	Transcriptional Misregulation
EGF	−9,14	0,00000001	MAPK, PI3K, Ras
PPARGC1A	−4,53	0,00000001	Chromatin Modification
FGF22	−2,17	0,00000001	MAPK, PI3K, Ras
CALML3	−1,45	0,02524279	Ras
CHAD	−3,19	0,00001052	PI3K
RASAL1	−5,81	0,00000001	Ras
WIF1	−12,16	0,00000001	Wnt

### Tumor-relevant gene expression signature in adenoid cystic carcinoma

The divergent gene expression pattern between tumor and non-tumor area of ACC showed, as demonstrated in Figure 3B and Table 2B, also a significant upregulation of *COMP*, *BMP7* and *MYB*. An upregulation is also found in matrix proteins such as *COL2A1* and *COL5A1*. As shown in Figure 4B, the analysis of the corresponding ACC and non-tumor samples showed an upregulation of *COMP*, *TLX1*, *MYB*, *BMP7*, *AMH*, *STMN1*, *BRIP1*, *ETVA*, *COL27A1*, *HIST1H3B*, *BMPR1B*, *FANCA*, *BMP8A*, *RASGRP1* and *RAD51*. In contrast, significantly downregulated, are *LRP2*, *ETV1*, *WIF1*, *EGF*, *FGF10*, *12*, *22*, *WNT5A*, *SIX1*, *IL19*, *ACVR1C*, *LEP*, *CHAD*, *FUT8* and *ANGPT1* (Table 3B).

### Tumor-relevant gene expression signature in adenocarcinoma NOS

The divergent gene expression pattern between tumor and non-tumor areas of the adenocarcinoma NOS also demonstrated a distinct upregulation of *COMP* as shown in Figure 3C and Table 2C. An upregulation is also found in matrix proteins like *COL11A1*, *COL1A1* and *FN1*.

As shown in Figure 4C, the analysis of the corresponding AC-NOS tumor and non-tumor samples showed a stronger upregulation of genes than down-regulation. Significantly upregulated genes included *COMP*, *SPP1*, *COL1A1*, *WNT7B*, *MMP9*, *FN1*, *ZIC2*, *HIST1H3B*, *IBSP*, *WNT2*, *ITGB6*, *HIST1H3G*, *FGFR4* and *COL1A2*. Downregulated genes were *WIF1*, *RASAL 1*, *CHAD*, *CALML3*, *FGF22*, *PPARGC1A*, *EGF*, *ETV1*, *PLCB4*, *IL19*, *LRP2*, *PROM1*, *RXRG*, *CACNB2* and *COL6A6* (Table 3C).

All AC-NOS showed an overexpression of COMP, in ACC all tumors showed an overexpression except one, in MEC there were two tumor samples that showed no expression. Thus, an overexpression of 44/47 (93.6%) was found.

### Genetic differences within tumor entities in regard to signaling pathways (advanced analysis)

**Figure 5:**
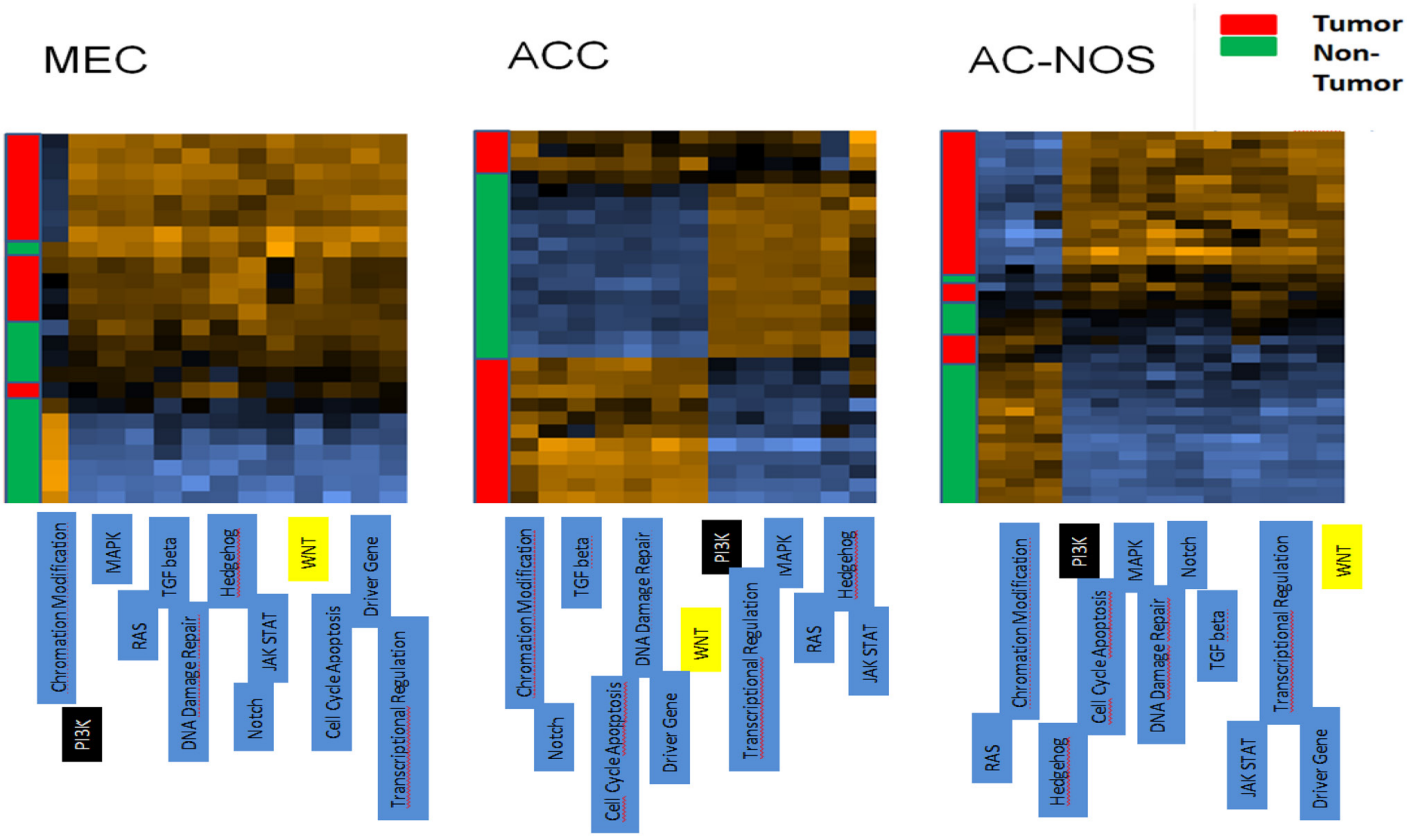
Cluster analysis of patient tumor samples and corresponding non-tumor of each tumor entity (MEC, ACC, AC-NOS). The tumor samples are arranged on the y-axis, the pathways are arranged on the x-axis. A gene expression higher than normal is shown in the heatmap brown, whereas a downregulated expression in comparison to normal is shown in blue. Yellow marked boxes indicate the Wnt pathway, black boxes the PI3K-pathway.

Most notably, the overall expression studies and a detailed analysis of the expression pattern of matching tumor versus non-tumor samples, followed by advanced pathway analysis, proved a prominent dysregulation of the Wnt signaling pathway in the three SGC entities (see Figure 5). Moreover, transcript upstream analysis demonstrated a pronounced activation of the PI3K pathway in AC-NOS and MEC, which is a main target of current cancer therapeutic strategies (see Figure 5).

## DISCUSSION

In the present study, a gene expression analysis of more than 700 genes in salivary gland carcinomas and their corresponding non-tumor tissue samples was performed. Salivary gland carcinomas of the three most common entities were considered. The gene expression profiles showed clear differences between tumor and non-tumor tissue across all entities. In addition, the entities showed a different gene profile when compared to one another. The Wnt signaling pathway and the PI3K signaling pathway were particularly striking in the individual examination of dysregulated genes and signaling pathways. These could represent a starting point for further research to deepen the understanding of the molecular genetic processes of salivary gland carcinomas and the development of new diagnostic and therapeutic options.

In recent decades, recurrent and therapy-relevant gene changes have been demonstrated for various organs. For example, a therapy with tyrosine kinase inhibitors in treatment protocols has been implemented in EGFR-mutated pulmonary carcinomas [13, 14]. EGFR-directed treatment strategies were introduced in colorectal adenocarcinomas wild-type for NRAS and KRAS [12] and BRAF inhibitor treatment finds application in BRAF V600E mutated malignant melanomas [16]. A structured evaluation of the genetics of the tumor tissue in a high number of salivary gland cancers has not been performed yet [15]. This is due on the one hand to the rarity of this tumor entity and on the other hand to the heterogeneity of the tumors in salivary glands. Grünewald et al. were able to carry out mutation analyses of 84 tumors in 13 subtypes of parotid carcinomas. Using a next-generation sequencing methodology, mutations could be shown in 22 different genes; mostly affected were *TP53*, followed by *RAS* genes, *PIK3CA*, *SMAD4* and members of the *ERB* family. However, these tumors driving genetic alterations were not found in MEC, ACC and AC-NOS [16].

Across all entities, it can be concluded from the data of this study that numerous genes of the Wnt signaling pathway are dysregulated in the examined salivary gland tumors (Tables 2 and 3). The strong and significant downregulation of *WIF1* is consistent between the three entities. The significantly reduced expression of the genes *PLCB4* (MEC and AC-NOS), *PLCB1* (MEC), *CAMK2B* (MEC) and *WNT5A* (MEC and ACC) was also found in the investigated entities (Table 3). Pathological activation of the Wnt signaling pathway in colon carcinomas and melanomas has been described more than 20 years ago. Further evidence can be found for prostate cancer, leukemia and medulloblastoma [17]. The importance of the Wnt signaling pathway for salivary gland tumors was also highlighted as early as 1988. From mouse models, it is sufficiently proven that a constant activation of the Wnt signaling pathway contributes to hyperplasia and oncogenesis of the salivary glands. An increased expression of the *WNT1* gene led to an increased incidence of breast and salivary gland tumors in mice [18]. In addition, it became clear that upregulation of the β-catenin gene (*CTNNB1*) and β-catenin target genes through inappropriate activation of transcription factors can trigger proliferation and de-differentiation of epithelial cells of the salivary glands and thus contribute to benign and malignant tumor formation [19–23]. The expression of further genes of the Wnt signaling pathway was downregulated in this study. These are *WNT5A*, which is strongly and significantly decreased in MEC and ACC. In a review by Tabatabai et al., it is stated that an increased expression of *WNT5A* had a tumor-inhibiting effect in various tumors such as neuroblastomas, breast carcinomas, thyroid carcinomas and leukemia [24]. *WIF1*, which was significantly downregulated in all entities, was recently reported in several human carcinomas, correlating with aberrant Wnt/β-catenin signaling, including salivary gland ACC by Wang et al. [25].

In all entities *COMP* was highly upregulated. Cartilage oligomeric matrix protein (COMP) is an extracellular matrix protein expressed via different tumors and contributes to the progression of various malignant diseases. COMP is a 524 kDa Ca2+-binding glycoprotein indicated primarily in cartilage tissue and plays an important role in the formation of the extracellular matrix. In addition, it regulates the activation and inhibition of the complement system, which is an important system of innate immunity as the first defense against invasion by microorganisms and altered cells. COMP (also called TSP-5) belongs to the thrombospondin family and is dependent on Ca2+ in its structure and function. Mutations in the Ca2+-binding region of COMP lead to skeletal dysplasia [26]. COMP has been shown in recent studies to be a promising new marker for breast cancer [27]. It was shown that up to 79% of malignant breast tumors express COMP more strongly and that the expression is significantly related to factors such as low survival rate, larger tumors *in vivo*, increased invasion *in vitro*, protection against stress-induced apoptosis, more frequent metastasis and the Warburg effect. The large tumor volume is not caused, as previously assumed, by COMP-induced extracellular matrix formation in the tumor stroma, as the tumor mass is mainly composed of tumor cells and less of stroma cells. COMP-expressing cells appear to have a greater capacity for proliferation, invasion and metastasis [27].

A high proportion of COMP in breast cancer and in salivary gland tumors may indicate an analogy of tumor structure and tumorigenesis. Triantafyllou et al. demonstrated parallels of the pathology of salivary and breast cancers with respect to the composition of myoepithelial cells, stromal components, analogues of mucosa-associated lymphoid tissue, steroid receptors and intraparenchymal cells of monocytic origin [28]. This results in interesting questions for future projects regarding the analogy of tumor development of the significantly more frequent breast cancers and salivary gland carcinomas.

In addition to COMP, all entities show dysregulated gene expression compared to normal tissue in numerous components of the PI3K pathway (Tables 2 and 3).

Particularly in AC-NOS, upregulation of a large number of genes that are transcriptional target genes for activation of the PI3K pathway became evident. Interestingly, these target genes include genes, coding for extracellular matrix proteins such as FN1, SSP1, COL1A1 or LAMC2. Studies have shown that PI3K plays an important role in growth regulation in both healthy and malignant degenerate cells of many tumors, and that genes of the PI3K/AKT pathway are the most frequently altered genes in human carcinomas [29]. This signaling pathway could also be suitable as a diagnostic agent and therapeutic target domain. Various PI3K inhibitors are currently undergoing clinical trials, in combination or monosubstance therapy, in solid and hematological cancers [30]. Due to the multitude of genetic changes within this signaling pathway (p110, p85, AKT, mTOR, PTEN, etc.) there would be various molecular targets for a therapy whose further research seems promising. In addition, it is important to identify the key elements of the signaling pathway in order to achieve the most effective therapy possible.

The demonstrated results suggested that in ACC, genes of inflammatory pathways were upregulated such as *TNF1* as wells as *interleukin 1* and *6 receptors* and mediators (e.g., *IRAK 2*, *3*, *IL1B*, *JAK3*, *STAT1*, *STAT4*) (Supplementary Figure 1C). This result seems unusual, as ACC has been described as less immunogenic in different studies [31]. ACCs showed a suppressed immune system in Tumor microenvironment in a study by Linxweiler. Characteristic were however the presence of M2-polarized macrophages and myeloid suppressor cells and a low Tumor mutational burden [31]. Especially macrophages release TNF and interleukin 1 and 6. Thus, the data do not inevitably contradict the aspect that ACC are generally immunologically cold.

Another interesting result was also that the entity of AC-NOS, which can histologically represent a very heterogeneous entity, showed a rather homogeneous group in the evaluation of the nanostring analysis.

Surely the largest limitation of the study is the low number of cases of the individual tumor entities. Multicentric studies should be pursued in order to be able to put the data presented here on a broader basis. Likewise, to limit it, this study involved only 3 entities, albeit frequent ones. No validation cohort could be measured due to the small number of cases of the individual tumors and in portions of insufficient tumor material. Corresponding measurements are being planned on a multicenter basis in subsequent projects. The PanCancer pathways covers a wide range of genes, there may be clinically relevant genes that were not assessed. The study does not address intratumoral heterogeneity. It would be conceivable that gene expression in the area of the invasion front differed from those in the solid tumor area. The status of the fusion genes, especially in ACC and MEC, were not known in the patients and were not included in the study. These could have an influence on the results. Further studies should investigate a correlation.

In the group of AC-NOS is an entity of adenocarcinomas, which cannot be divided further. Thus, certain heterogeneity of the tumors is assumed. It may thus be difficult to identify a consistent pattern of gene expression in this entity.

In future studies, the results of the gene analytical studies should also be correlated with clinical factors such as survival rates, response to therapy, metastasis or recurrence rates.

Despite these limitations, the present work contributes to the knowledge of the genetic mechanisms of this rare form of tumor to a large extent and offers a multitude of starting points for more intensive research due to the high number of genes analyzed.

## MATERIALS AND METHODS

### Patient specimens and clinical data

The analysis was conducted according to the Declaration of Helsinki on biomedical research involving human subjects. A total of 60 PGC patients with MEC (*n* = 20), ACC (*n* = 20) and AC-NOS (*n* = 20) were applied to the study. All patients were treated for PGC with primary surgery between 1990 and 2014 at the Department of Otorhinolaryngology, Head and Neck Surgery, University Hospital of Cologne, Germany. All specimens were obtained from the tissue bank of the Institute for Pathology at the University Hospital of Cologne (Cologne, Germany) and were used in accordance with the policies of the local institutional review board of the hospital. All specimens used for gene expression profiling were reviewed and selected by two experienced pathologists (J. M., U. D.). The data that support the findings of this study are available from the corresponding author upon reasonable request.

Patients who were staged clinically as being negative for lymph node metastasis (cN0) had been treated with selective neck dissection at least for levels I–III. Patients with clinically positive cervical lymph node status (cN1-3) had received a modified radical neck dissection. In cases of high-grade carcinoma of MEC (G3 or G4 based on the AJCC Cancer Staging Manual) incomplete resection margins, loco-regional neck lymph node metastasis and perineural invasion adjuvant RT were additionally administered.

Tumor and corresponding non-tumor areas from formalin-fixed and paraffin-embedded tissues were macrodissected from formalin fixed and paraffin-embedded tissues and used for extraction. After RNA quantification, 94 matching tumor—non-tumor samples from a total of 47 cases (Supplementary Table 1 and Table 1) including 14 cases of ACC, 20 cases of AC-NOS and 13 patients suffering from MEC from which sufficient RNA was isolated—were chosen for expression profiling of oncogenic and tumor suppressor genes by means of NanoString’s nCounter technology.

### Microdissection and RNA isolation

Sections were prepared and stained with hematoxylin and eosin (HE) according to the standard protocol. Histological examination using HE-sections provides a targeted assessment of the areas that have the highest—at least 70–80%—tumor content. Corresponding to the sample hematoxylin and eosin staining, tumor and non-tumor areas were collected via macrodissection as described before the sections were scraped off with a scalpel and collected into plastic tubes [32].

The total RNA was then isolated using the RNA FFPE kit from Promega on the Maxwell^®^ 16 LEV (Promega AS1260, Heidelberg) according to the manufacturer’s instructions. RNA concentration was quantified by the QuantiFluor^®^ RNA System, as recommended by the supplier (Promega, E6090).

After concentrating the RNA to 150 ng in 5 μL by means of a speed vac, the samples were taken for the Nanostring analysis.

### N-Counter hybridization and gene expression analysis

150 ng RNA from each specimen were used for N-Counter hybridization technology using the PanCancer Pathways Panel (Nanostring, Seattle, WA, USA) (Supplementary Table 2) following the manufacturer´s recommendations. Briefly, the RNA extracts to be tested are denatured at 85°C and incubated with the reporter and capture probes in a thermocycler overnight (12 hours) at 65°C. Fluorochrome barcoded hybrids were captured and purified using the Prep Station platform (Nanostring) and subsequently quantified by the Digital Analyzer (Nanostring).

For normalization of expression profiles, 35 housekeeping genes were selected using the GeNorm algorithms (Supplementary Table 3). Expression profiles were then studied using the NSolver 4 software from Nanostring. The analysis was carried out according to the manual of the NSolver 4 software (Gene Expression Data Analysis Guidelines, MAN-C0011-04, https://www.nanostring.com/support/data-analysis/nsolver-data-analysis-support). Background signals, determined as the mean values of all system negative controls, were subtracted from probe signals. Hierarchical clustering was performed using the algorithm of Euclidean distance calculation. For pathway analysis, either the Nanostring pathway annotation or GO analysis was carried out. In addition, we studied gene expression of the matching tumor and non-tumor samples, considering only genes that were divergently expressed more than twofold in tumor vs non-tumor of greater than 66.7% of the respective PGC entity.

## CONCLUSIONS

Gene expression analysis revealed a clear difference in the gene expression profile between tumor tissue and non-tumor tissue in MEC, ACC and AC-NOS. In addition, the entities can be distinguished from each other by their differential gene profile. In addition to a large number of dysregulated genes, the expression pattern of individual genes manifests a cross-entity dysregulation in the Wnt signaling pathway, in particular a downregulation of WIF1 and WNT5A. In the PIK3 signaling pathway, an upregulation of *COMP* is particularly noticeable. In particular, these signaling pathways could offer possibilities for personalized therapy in the future.

## SUPPLEMENTARY MATERIALS




