# Supplementary material for: Expression profiling on subclasses of primary parotid gland carcinomas
Source: Oncotarget. 2020 Nov 10;11(45):4123–37. doi: 10.18632/oncotarget.27797 (PMC7665229; doi:10.18632/oncotarget.27797)
Supplement: Supplementary file 1 [file oncotarget-11-4123-s001.pdf]

Expression profiling on subclasses of primary parotid gland carcinomas

SUPPLEMENTARY MATERIALS

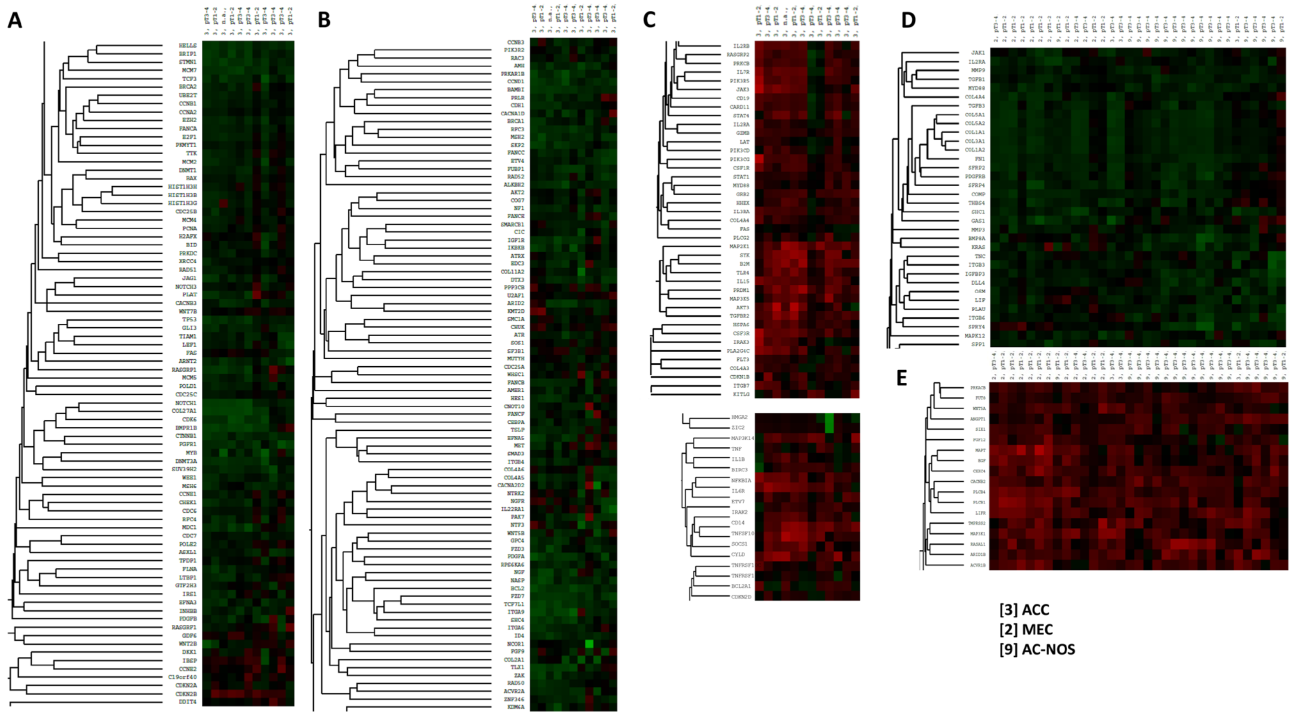

Supplementary Figure 1: Extract from the hierarchical cluster analysis of all 47 patient samples of tumor tissue and corresponding non-tumor tissue. Detailed comparison of the expression profiles of MEC, ACC and AC-NOS confirmed that the differences in expression clusters of ACC in comparison to MEC and AC-NOS (**A**–**C**), whereas MEC and AC-NOS differ only slightly from each other (**D**–**E**). Importantly in ACC, genes of inflammatory pathways were upregulated such as TNF1 as well as interleukin 1 and 6 and mediators (e.g., IRAK 2, 3, IL1B, JAK3, STAT1, STAT4) (C). In MEC and AC-NOS, for example, the subunits of phospholipase C, D, FGF12 and the chemokine CXXC4 were highly expressed (E).

Supplementary Table 1: Detailed chart of the clinical characteristics of the included patients. See Supplementary Tables 1 and 2

Supplementary Table 2: Listing of the genes investigated in the PanCancer pathway. See Supplementary Tables 1 and 2

Supplementary Table 3: Listing of the housekeeping genes included in the PanCancer pathway

Gene	Acession No.	Signal	Housekeeping Selection	geNorm SD after normalisation
ZNF384-mRNA	NM_133476.3	300	1	0.195
DDX50-mRNA	NM_024045.1	1185	2	0.218
PIK3R4-mRNA	NM_014602.1	3620	3	0.231
HDAC3-mRNA	NM_003883.2	1455	4	0.222
SAP130-mRNA	NM_024545.3	3090	5	0.256
TMUB2-mRNA	NM_024107.2	1485	6	0.25
CNOT4-mRNA	NM_001190848.1	795	7	0.242
TLK2-mRNA	NM_006852.2	2335	8	0.218
SLC4A1AP-mRNA	NM_018158.2	980	9	0.218
TRIM39-mRNA	NM_021253.3	3140	10	0.232
PRPF38A-mRNA	NM_032864.3	335	11	0.292
DHX16-mRNA	NM_001164239.1	2490	12	0.257
C10orf76-mRNA	NM_024541.2	3750	13	0.288
FCF1-mRNA	NM_015962.4	1022	14	0.306
SF3A3-mRNA	NM_006802.2	2060	15	0.312
ACAD9-mRNA	NM_014049.4	1935	16	0.303
EDC3-mRNA	NM_001142443.1	925	17	0.282
AGK-mRNA	NM_018238.3	816	18	0.32
MTMR14-mRNA	NM_022485.3	720	19	0.28
ZNF143-mRNA	NM_003442.5	925	20	0.311
MRPS5-mRNA	NM_031902.3	390	21	0.301
VPS33B-mRNA	NM_018668.3	2140	22	0.296
AMMECR1L-mRNA	NM_001199140.1	3564	23	0.318
DNAJC14-mRNA	NM_032364.5	1166	24	0.327
FTSJ2-mRNA	NM_013393.1	1435	25	0.35
EIF2B4-mRNA	NM_172195.3	1390	26	0.355
TTC31-mRNA	NR_027749.1	2720	27	0.358
CNOT10-mRNA	NM_001256741.1	1962	28	0.359
GPATCH3-mRNA	NM_022078.2	1685	29	0.36
ERCC3-mRNA	NM_000122.1	1950	30	0.363
PIAS1-mRNA	NM_016166.1	1870	31	0.365
ZC3H14-mRNA	NM_001160103.1	2690	32	0.404
ZKSCAN5-mRNA	NM_014569.3	3688	33	0.396
RBM45-mRNA	NM_152945.2	1080	34	0.399
COG7-mRNA	NM_153603.3	1492	35	0.403
USP39-mRNA	NM_001256725.1	806	*discarded*	0.474
ZNF346-mRNA	NM_012279.2	2260	*discarded*	0.501
NOL7-mRNA	NM_016167.3	335	*discarded*	0.577
NUBP1-mRNA	NM_001278506.1	304	*discarded*	0.603
CC2D1B-mRNA	NM_032449.2	4182	*discarded*	0.688
